# Digital health in clinical pharmacy practice: transforming precision medicine and pharmacometrics in Africa

**DOI:** 10.3389/fphar.2026.1924654

**Published:** 2026-08-25

**Authors:** Sumaiah J. Alarfaj, Engy Wahsh, Khaled Saad, Eman S. Sawan

**Affiliations:** 1 Department of Pharmacy Practice, College of Pharmacy, Princess Nourah bint Abdulrahman University, Riyadh, Saudi Arabia; 2 Clinical Pharmacy Department, Faculty of Pharmacy, October 6 University, Giza, Egypt; 3 Department of Pediatrics, Faculty of Medicine, Assiut University, Asyut, Egypt; 4 Department of Clinical Pharmacy, Faculty of Pharmacy, Badr University in Cairo (BUC), Badr, Egypt

**Keywords:** Africa, artificial intelligence, clinical decision support systems, clinical pharmacy, digital health, electronic health records, model-informed precision dosing, pharmacogenomics

## Abstract

African nations still struggle with several healthcare challenges such as high prevalence of communicable and non-communicable diseases, underdeveloped healthcare systems, lack of healthcare personnel, and uneven distribution of quality healthcare services. Resource constraints and a fragmented healthcare system make the aforementioned challenges even more difficult to solve. However, digital health technology and artificial intelligence (AI) can be viewed as revolutionary instruments that may help to overcome the mentioned challenges and enhance healthcare practices in Africa. The present article provides a review of how digital health technologies and AI influence clinical pharmacy practice and contribute to precision medicine and pharmacometrics in African nations. To do that, a literature search has been performed using the PubMed, Scopus, and Web of Science electronic databases for relevant articles about digital health, AI, clinical pharmacy, precision medicine, pharmacometrics, and healthcare delivery in Africa. Healthcare technologies, such as telemedicine, mobile health apps, electronic health records, and clinical decision support systems powered by AI, are becoming more pervasive and widening the scope of work for clinical pharmacists through their contribution to medication optimization, therapeutic monitoring, and evidence-based clinical decision-making. Their application within precision medicine allows creating an individual treatment strategy considering the genetic and clinical features of the patients. At the same time, pharmacometric approaches, especially pharmacokinetics and pharmacodynamics modeling and model-informed precision dosing, help to optimize drug prescription and therapy outcomes for different African populations. The present article provides a review of how integrating digital health technologies and AI into clinical pharmacy practice could be a promising approach for enhancing precision medicine in Africa. The review also explores their implementation challenges and future opportunities for reasonable healthcare delivery.

## Graphical Abstract

**Figure:**
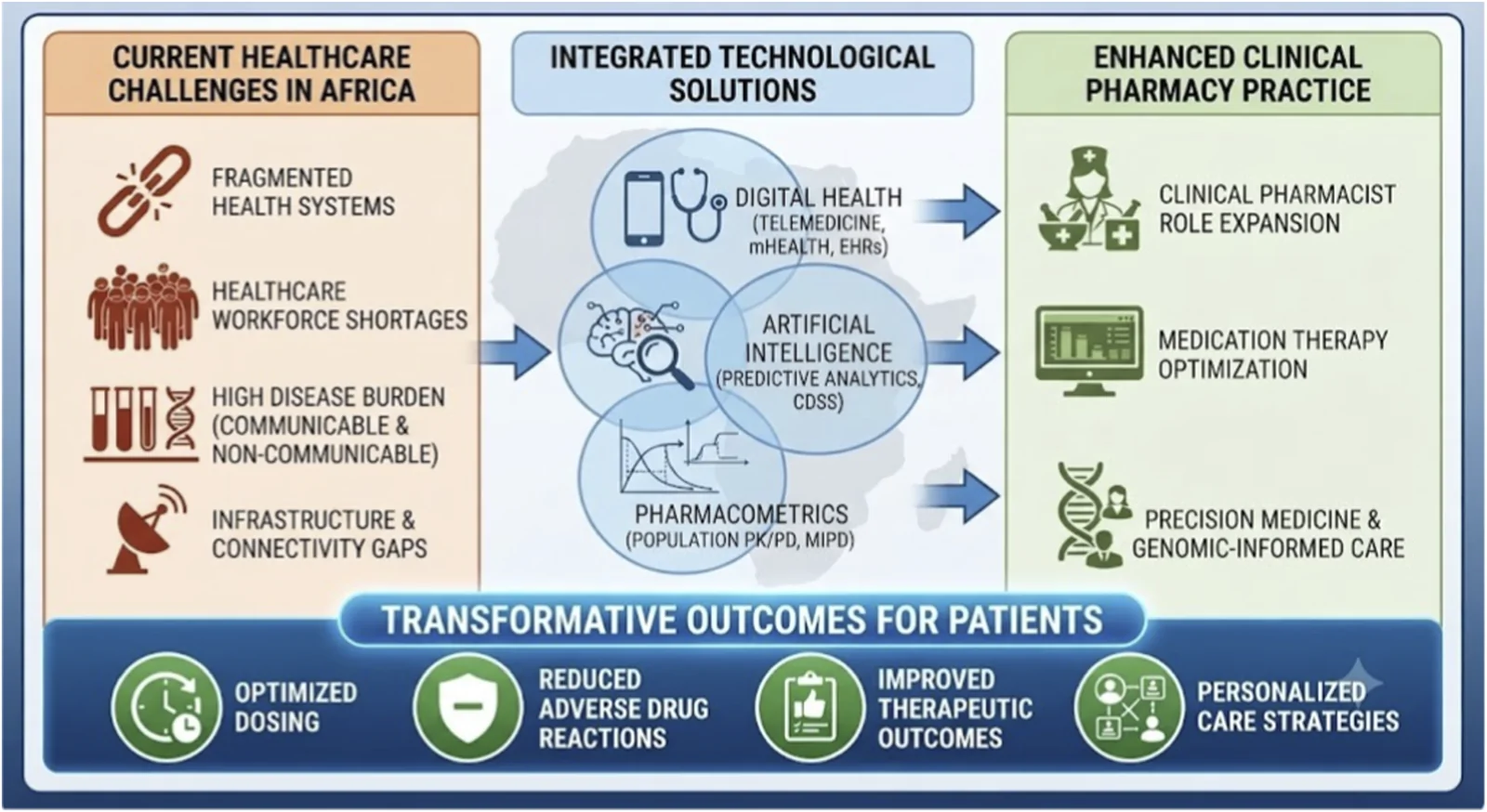

## 1 Introduction

Healthcare systems globally are experiencing a revolution because of advances in digital health, artificial intelligence (AI), and evidence-based clinical decision-making (Hussain et al., 2022). The advent of digital health, which is made up of e-health records, telemedicine, mobile applications, wearable technologies, and AI-powered clinical systems, has been a key component that has enabled modernization of healthcare delivery through improved access to services, diagnostic accuracy, better clinical workflows, and more effective decision-making in therapeutic interventions (Alarfaj et al., 2024; Singla et al., 2025). Additionally, artificial intelligence has added provocation to this trend through advanced analysis, prediction models, and interpretation of data related to healthcare in real time. Applications of AI technology in healthcare include diagnostic tests, risk stratification, clinical decision support, optimization of treatments, and personalization of treatments. This technology is facilitating a change from reactivity in healthcare delivery to proactivity and personalized care in the sector (Gouripur, 2024).

In addition to the digital transformation of the healthcare delivery process, the profession of clinical pharmacists has seen a transition from a traditional one of just providing medicines to patient-centered clinical practice. Clinical pharmacists are currently playing very important roles in optimizing medications, pharmacovigilance, antimicrobial stewardship, therapeutic drug monitoring, and decision-making in a multi-disciplinary environment (Alshahrani et al., 2026; Spiro, 2019). The integration of digital health and artificial intelligence into clinical pharmacy practice opens up vast possibilities for improving medication management and therapeutic outcomes. The digitalization of healthcare provides clinical pharmacists with increased access to clinical information about the patient, while artificial intelligence-based systems help to predict risks, monitor medication safety, and optimize therapies (Owaidhah et al., 2025).

In parallel, precision medicine is developing into an innovative paradigm of modern medicine. Precision medicine involves personalizing the preventive and treatment approaches according to the variations between individuals concerning their genes, environment, lifestyle, and other factors. In contrast to traditional population-based approaches to therapy, precision medicine aims at delivering more targeted and effective treatments (Sarwar, 2023). Pharmacometrics also contributes to precision medicine by offering quantitative approaches to individualized dosing and treatment. Using mathematical modeling of pharmacokinetics, pharmacodynamics, and responses to treatment, pharmacometric approaches help deliver model-informed precision dosing and improved decisions regarding therapy. Such approaches are considered to be an integral part of personalized medicine (Preijers et al., 2024).

In Africa, the combination of digital health, AI, precision medicine, and pharmacometric advancements offers numerous opportunities for overcoming some of the healthcare obstacles. In particular, African countries still struggle with the following problems: shortage of the healthcare workforce, access to specialists, healthcare fragmentation, lack of resources, and a significant burden of communicable and non-communicable diseases. This creates high demand for innovative and scalable healthcare solutions (Adeagbo et al., 2026; Ogunye et al., 2024). Moreover, African populations provide an enormous amount of genetic diversity, which gives great possibilities for research in genomics and precision medicine. At the same time, populations of Africa are considerably underrepresented in the global genomic, pharmacometric, and biomedical databases. It hinders the usability of existing models and tools in precision medicine in the African healthcare environment (Sibomana, 2024).

Although the importance of digital health and AI increases all over the world, there is little scientific literature that has considered the role of both concepts in combination for clinical pharmacy practice, precision medicine, and pharmacometric application in the African healthcare environment. Existing research deals with the described areas individually. Therefore, this review aims to critically evaluate the transformative role of digital health and artificial intelligence in clinical pharmacy practice and their contributions to precision medicine and pharmacometric applications in Africa. It further explores current opportunities, implementation challenges, and future directions for advancing data-driven, personalized, and equitable healthcare delivery across African healthcare systems.

## 2 Methods

This study was performed as a narrative literature review to critically assess the potential of digital health and artificial intelligence (AI) in clinical pharmacy practice and their contribution to the development of precision medicine and pharmacometric applications in Africa. An extensive literature search was conducted using databases such as PubMed, Scopus, Web of Science, and Google Scholar, together with WHO and CDC-Africa reports and policies. The literature search covered articles published between January 2010 and May 2026 using keywords such as digital health, artificial intelligence, clinical pharmacy, precision medicine, pharmacogenomics, pharmacometrics, model-informed precision dosing, and Africa.

Included sources for the literature review included original research articles, systematic/narrative reviews, clinical guidelines, consensus statements, policy papers, and reports relevant to digital health, AI, clinical pharmacy practice, precision medicine, and pharmacometric applications in Africa. Scientifically poor research studies, duplicate studies, editorials, and non-peer-reviewed articles were excluded. Articles were assessed for eligibility based on the review of their titles, abstracts, and entire texts. Relevant data from articles were collected and analyzed using the thematic analysis technique.

## 3 Digital health and artificial intelligence landscape in Africa

There is an ongoing digital revolution happening in the healthcare sector in Africa, which is facilitated by the growing use of mobile devices, increasing availability of internet connectivity, and investments in innovations in health technology. Digital health has become a vital tool that can be used to tackle some problems faced by healthcare systems across the African continent, such as poor access to healthcare, lack of healthcare personnel, fragmentation of health systems, and inequalities in healthcare provision. The above-mentioned issues affect African rural areas and poorly served communities, and therefore it is difficult to receive specialized services there. This is why digital technologies provide innovative solutions to these issues (Badaru and Mphahlele, 2023).

The term “digital health” includes various technologies, such as telemedicine, mobile health (mHealth), electronic health records (EHRs), remote monitoring, and artificial intelligence (AI)-enabled healthcare. There is an increasing adoption of those technologies in African healthcare systems to improve the provision of healthcare services, optimize clinical workflows, conduct disease surveillance, and make decisions based on the data gathered. Countries such as Kenya, Rwanda, Nigeria, South Africa, and Egypt have shown their success in introducing digital health innovations using telemedicine applications, mobile health technologies, artificial intelligence-based diagnostics, and disease surveillance systems (Oladosu et al., 2025).

### 3.1 Digital health transformation in Africa

The use of digital health technologies in Africa is primarily attributed to the widespread use of mobile technology. The penetration of mobile phones in Africa has greatly improved, thus providing great opportunities to use mobile technology as an efficient platform for health services delivery. The use of mobile applications has been particularly effective in the management of medication adherence, education of patients, disease monitoring, and patient-physician communication. This technology has facilitated the improvement of healthcare engagement and continuity of healthcare delivery in rural and other disadvantaged areas (Huisman et al., 2022).

While mHealth applications have led to increased accessibility of healthcare, improved medication compliance, and greater engagement with patients, issues related to security, interoperability and software quality pose serious obstacles for implementation. Thus, future developments in mHealth applications must be focused on the evidence-based application development process and clinical validation as the key factors that will provide sustainable changes in the healthcare delivery processes (Jakob et al., 2022).

Telemedicine is another innovative digital technology that has made tremendous contributions to African healthcare. It provides the possibility of remote consultations, referrals to specialists, and continuity of care for those who live in different regions (Omaghomi et al., 2024). Telemedicine platforms have removed obstacles that were caused by the distance factor and the lack of specialists, thus facilitating access to healthcare for people living in rural and other disadvantaged areas. Rwanda, Kenya, and South Africa have demonstrated some advances in the implementation of telemedicine solutions and digital healthcare delivery (Agbeyangi and Lukose, 2025).

With the rapid rise of telemedicine in Africa, the sustainability of the telemedicine services will require enhanced digital infrastructure development, secure connection to the Internet, and regulation of cross-border healthcare delivery and reimbursement. Moreover, the current differences between the urban and rural healthcare organizations’ level limit the possibility of providing telemedicine services. The future implementation efforts thus should focus on infrastructure development and healthcare personnel training (Castillo et al., 2023).

Electronic health records and digital data management systems have also started to be implemented for the improvement of data management and documentation. Though adoption still varies in African healthcare systems, EHRs offer an important basis for building better health information systems and improving healthcare delivery (Harerimana et al., 2025). Moreover, remote monitoring systems are becoming a popular means for managing chronic diseases and providing long-term monitoring of patients. These systems allow continuous monitoring of patients, early intervention, and prevention of unnecessary hospitalization. Their application in healthcare delivery will help manage chronic diseases better and increase patient-centeredness (Mbanugo, 2025; Tegegne et al., 2026).

Electronic health record systems have become critical elements of today’s healthcare system, but fragmented adoption, lack of interoperability, and varying data quality levels limit their benefits in most African healthcare systems. Data governance models, interoperability, and continued government funding will be crucial in fully utilizing the benefits of EHR platforms and advancing precision medicine using artificial intelligence technology (Harerimana et al., 2025).

### 3.2 Adoption of artificial intelligence in healthcare

The increasing role of artificial intelligence in the healthcare industry is linked to its ability to work with large data sets, automate clinical processes, and make decisions based on evidence. The scope of AI implementation in African healthcare is widening, affecting various areas, including disease diagnostics, medical imaging, predictive analysis, and clinical decision support systems (Behara et al., 2022). AI-driven diagnostic solutions show good applications in the area of disease screening, medical image analysis, and early diagnosis of diseases. Machine learning algorithms can help increase diagnostic precision, accelerate clinical processes, and make healthcare delivery more efficient. These features will be especially useful in those settings that lack specialist resources. AI-assisted diagnostics have become relevant in African countries like South Africa and Egypt, especially in radiology and disease detection applications. Another significant use case of AI in healthcare involves AI-powered clinical decision support systems (CDSS). These systems assist clinicians in assessing patient-specific information, detecting clinical risks, and designing treatment plans. Enhanced by AI, CDSS could help with risk prediction, treatment optimization, and medication safety (Elhaddad and Hamam, 2024).

### 3.3 Opportunities for digital innovations in African healthcare

There are many opportunities associated with digital health and AI applications in solving healthcare challenges in African healthcare systems. One of the most prominent opportunities pertains to the issue of enhancing access to care for rural and vulnerable population segments. Telemedicine, mHealth, and remote monitoring technologies can help overcome geographical barriers to health and care continuity (Alaran et al., 2025). Moreover, the digital innovations will facilitate improvements in the operational efficiency of healthcare systems through better management of workflows, enhanced patient data management, and care coordination. EHRs and integrated health information systems will improve health services delivery as they provide timely access to clinical information (Oladosu et al., 2025).

AI can further enhance those opportunities due to predictive analytics, resource optimization, and healthcare planning driven by big health data analysis. AI extends these possibilities further by means of predictive analytics, resource optimization, and data-driven healthcare management. The ability of AI to process vast amounts of health data and make predictions on risks and proactively implement healthcare measures can help substantially in making healthcare systems more responsive and optimizing resource usage in environments where resources are scarce (Ibeneme et al., 2022).

Digital surveillance systems have gained much significance in terms of disease surveillance and outbreak response as well. The main function of such systems is disease surveillance, outbreak identification, and epidemiology monitoring. Such systems are especially important for epidemic preparedness and response in African nations (Achieng and Ogundaini, 2024; Maddah et al., 2023).

### 3.4 Current barriers and system-level challenges

Although great advancements have been made in the field, many barriers still exist that hinder the deployment of digital health and AI across the continent. The problem of limited infrastructure remains one of the most important issues. Many healthcare facilities still suffer from a lack of electricity, poor internet connectivity, and poor digital infrastructure (Nnaji, 2024).

Additional barriers include fragmentation of health information systems, lack of interoperability, financial issues, and lack of a skilled workforce in digital health, health informatics, and AI. Insufficient access to quality healthcare data also affects the effectiveness and scalability of AI-enabled systems (Omaghomi et al., 2024).

Barriers in regulation and governance make the process even more difficult, especially those related to data privacy, cybersecurity, and AI governance. These barriers will need to be addressed through investments in digital infrastructure, better health information systems, and workforce development, among others. Improving these building blocks will be critical for the success of the digital health revolution in Africa (Oyenuga, 2024). The major digital health and AI technologies enhancing healthcare delivery in Africa, with their main healthcare applications, potential benefits, major challenges, and representative examples, are summarized in Table 1.

**TABLE 1:** Major digital health and AI technologies transforming healthcare delivery in Africa.

Technology/application	Key healthcare applications	Potential benefits	Major challenges	Examples in Africa	Key references
Telemedicine	Remote consultations, specialist referrals, follow-up care	Improved healthcare access, reduced geographic barriers	Poor connectivity, infrastructure limitations	South Africa, Kenya, Nigeria, Ghana	Agbeyangi and Lukose (2025), Cuadros et al. (2024)
Mobile health (mHealth)	Medication reminders, patient education, maternal health, disease monitoring	Improved adherence, patient engagement	Limited smartphone access, internet costs	Kenya (M-TIBA), Ghana, Nigeria	Bene et al. (2024), Hinneh et al. (2025)
Electronic health records (EHRs)	Clinical documentation, patient data management	Improved workflow, reduced errors	High implementation cost, interoperability issues	South Africa, Kenya, Ethiopia	Werner et al. (2023)
Remote monitoring systems	Chronic disease follow-up, therapeutic monitoring	Early intervention, reduced hospital visits	Device cost, data integration issues	South Africa, Rwanda, Kenya	Mbanugo (2025), Tegegne et al. (2026)
AI diagnostics	Medical imaging, disease screening	Faster diagnosis, improved accuracy	Limited datasets, algorithm bias	South Africa, Egypt, Ghana	Townsend et al. (2023), Topol (2019)
AI clinical decision support	Risk prediction, treatment optimization	Improved decision-making, enhanced safety	Data quality issues, implementation complexity	Emerging in South Africa, Kenya, Nigeria	Elhaddad and Hamam (2024)
Digital surveillance systems	Disease tracking, outbreak monitoring	Early detection, improved public health response	Data fragmentation, weak reporting systems	Africa CDC, Rwanda, Nigeria, continental systems	Achieng and Ogundaini (2024), Maddah et al. (2023)

### 3.5 International and African guidelines supporting digital health and artificial intelligence

The successful implementation of digital health and artificial intelligence (AI) in clinical pharmacy and precision medicine requires good governance regulations and evidence-based execution guidelines (Furukawa et al., 2026). Over the last 10 years, there have been a number of strategic frameworks developed by different international and regional organizations aimed at ensuring safe, ethical, and effective integration of digital technologies in healthcare. This has been very helpful in developing digital health strategies in many parts of the world and specifically in Africa through the promotion of patient safety and data security while promoting equity in healthcare delivery (Selvaraj et al., 2022).

One of the best strategic frameworks for implementing digital health in healthcare delivery is provided by the WHO Global Strategy on Digital Health 2020–2025. In this framework, emphasis is placed on developing a national digital health ecosystem, improving digital literacy among healthcare professionals, and promoting the evidence-based adoption of digital technologies. Most importantly, in this framework, it is stressed that there should be integration of digital health into routine healthcare delivery rather than implementing isolated technological solutions. This framework has guided national digital health policies across many African countries, the matter that enhanced investments in telemedicine, electronic health records (EHRs), and mobile health (mHealth) platforms (World Health Organization, 2021b).

While the WHO Strategy supports digital transformation of the health system in any country worldwide, the Africa CDC Digital Transformation Strategy (2023–2030) targets specific health needs and challenges facing African nations. This Strategy deals with improving digital infrastructure, disease surveillance, information exchange, using artificial intelligence in public health services, and digital health workforce development. Through fostering collaboration between different digital platforms, this strategy aims to reduce differences in accessing healthcare and being prepared to respond to infectious diseases and other health emergencies (Africa Centres for Disease Control and Prevention, 2023).

Despite the increasing uptake of digital health solutions on the continent, the regulatory framework for health apps is disjointed. As pointed out in a scoping review by Bene et al., the current regulations concentrate mainly on software quality, cybersecurity, interoperability, data privacy, and clinical safety, but lack comprehensive regulatory frameworks for health apps in many Sub-Saharan African countries. The need for harmonized regulation was strongly expressed by the authors (Bene et al., 2024; World Health Organization, 2021b).

Along with the digital health strategies, WHO Guidance on Ethics and Governance of Artificial Intelligence for Health provides internationally established ethics and governance principles that will ensure safe use of artificial intelligence in the area of healthcare. Transparency, explainability, fairness, privacy, human oversight, and reasonable access to technologies that are based on artificial intelligence are the key principles. These ethical principles are especially important considering the idiosyncrasies of the African healthcare systems where algorithmic bias, insufficient local databases, and regulatory capacity in Africa may influence the safe deployment of AI-based clinical decision support systems World Health Organization, 2021a).

From a technological viewpoint, the development of interoperability standards, such as Fast Healthcare Interoperability Resources (FHIR) of the Health Level Seven International (HL7) Organization, has become a crucial tool for ensuring safe sharing of patient information between healthcare organizations. Moreover, the international regulatory activities, initiated by various organizations, such as the International Medical Device Regulators Forum (IMDRF), offer guidance on how to evaluate, validate, and regulate software-based medical devices and clinical applications supported by artificial intelligence (Sah and Ramesh, 2025).

Although these international and regional regulations have contributed significantly to digital health adoption in Africa, the use of the proposed guidelines is uneven among African countries. Rwanda, Kenya, South Africa, and Egypt are the countries that have made considerable efforts to develop national digital health policies and increase electronic health records usage and integration of telemedicine services in healthcare provision. However, many low-resource countries face challenges associated with insufficient digital infrastructure, fragmented health information systems, financial investment, human resources, and regulatory capabilities.

In summary, there are solid strategic guidelines that can help realize the process of digital transformation within the African healthcare system. However, success in the long run relies mainly on consistent government support, more investments in digital infrastructure, development of locally adapted AI algorithms, regulatory framework harmonization, and capacity building among healthcare providers, including clinical pharmacists. Strengthening these implementation strategies will be crucial for translating policy recommendations into measurable developments in patient care, medication safety, and precision medicine across the continent. Guidelines Supporting Digital Health and AI Implementation in Africa are illustrated in Table 2.

**TABLE 2:** Major international and regional guidelines supporting digital health and AI implementation in Africa.

Guideline	Organization	Main focus	Relevance to Africa
WHO global strategy on digital health (2020–2025)	WHO	Digital health governance, interoperability, workforce	National digital health implementation
Ethics and governance of AI for health	WHO	Ethical AI, transparency, accountability	Safe AI deployment
Digital transformation strategy (2023–2030)	Africa CDC	Digital infrastructure, surveillance, AI	African regional strategy
HL7 FHIR	HL7	Health information interoperability	EHR integration
IMDRF SaMD	IMDRF	Regulation of AI software	Clinical decision support systems
Regulatory standards for health apps	JMIR Scoping Review	Regulation of mHealth applications	Safe implementation of health apps

Key international and regional guidelines supporting the implementation of digital health and artificial intelligence in African healthcare systems, highlighting their primary objectives and relevance to healthcare delivery across the continent. WHO, world health organization; AI, artificial intelligence; HL7 FHIR, health level seven fast healthcare interoperability resources; IMDRF, international medical device regulators forum; SaMD, software as a medical device; EHR, electronic health record.

## 4 Role of clinical pharmacy in digital health

With the fast-growing adoption of digital health technologies and artificial intelligence (AI) in clinical practice, there are significant changes in the role of the clinical pharmacist in many countries worldwide, including Africa (Alam et al., 2025). Previously, clinical pharmacists performed important roles in medication management, medication safety, therapeutic drug monitoring, and patient education. With the adoption of digital health technologies within healthcare organizations, clinical pharmacists began applying technological solutions to achieve optimized medication management and positive patient outcomes (Alqahtani et al., 2025).

**FIGURE 1:**
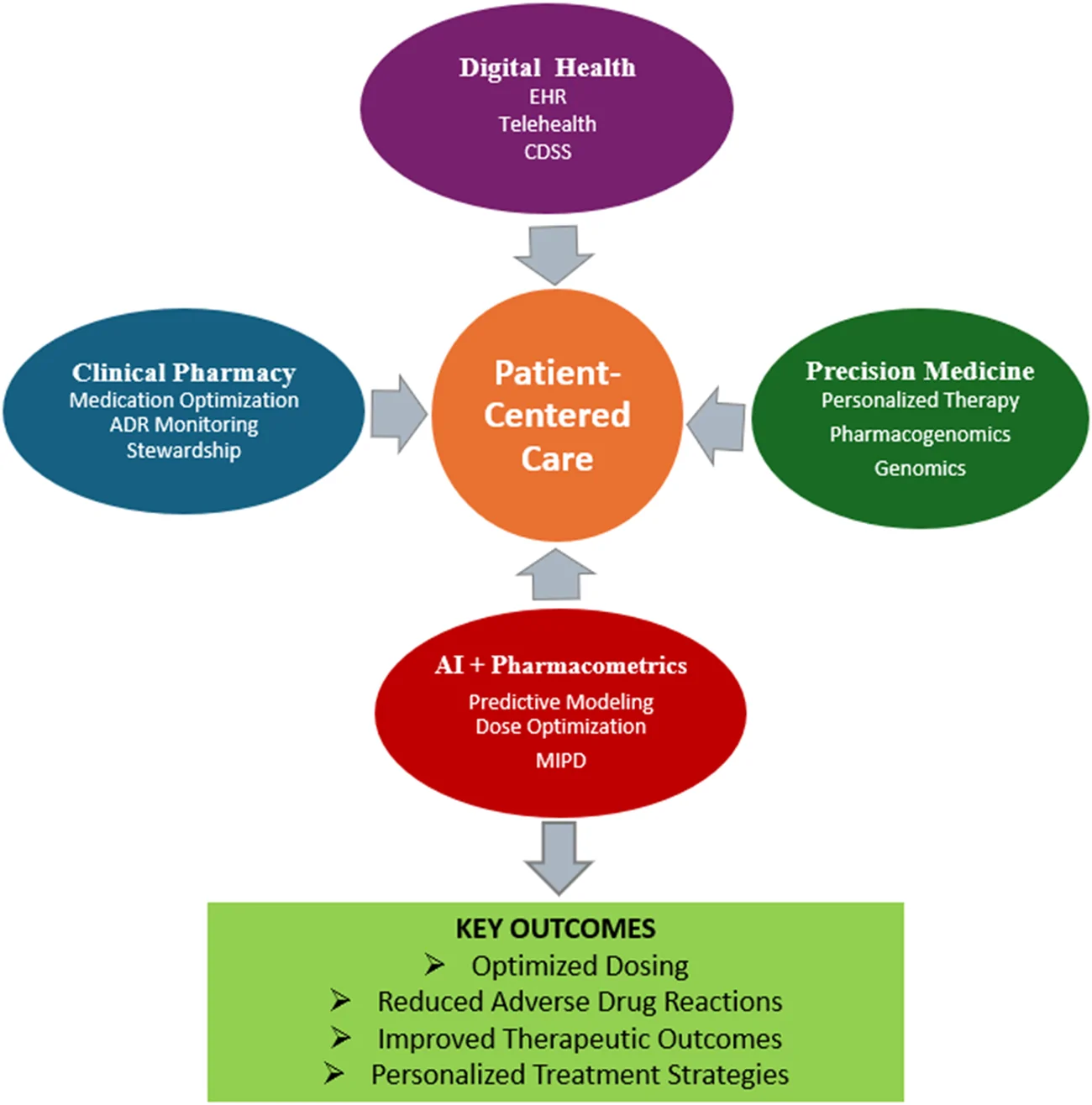
Conceptual framework of clinical pharmacy, digital health, artificial intelligence, precision medicine, and pharmacometrics. This framework illustrates how digital health, AI, precision medicine, pharmacometrics, and clinical pharmacy collectively support patient-centered care through optimized therapy, reduced adverse drug reactions, and improved therapeutic outcomes.

The use of digital health technologies such as electronic prescribing systems, clinical decision support systems (CDSS), electronic health records (EHRs), remote monitoring technologies, and AI-enabled analytics has allowed increasing opportunities for clinical pharmacists to perform patient care activities in a more efficient and data-driven way. Digital health technologies provide additional options for medication optimization, medication safety, pharmacovigilance, and antimicrobial stewardship activities. The adoption of digital models of healthcare delivery by healthcare organizations contributes to further evolution of the role of clinical pharmacists from traditional medication dispensing to clinical decision-making and therapeutic management (Awala and Olutimehin, 2024; Topol, 2019). The conceptual framework shown in Figure 1 summarizes the interactions among these key components within African healthcare systems.

### 4.1 Medication therapy optimization

Optimization of medication therapy is one of the core functions of a clinical pharmacist. Medication therapy optimization implies ensuring the use of appropriate drugs in the right dose and duration of treatment to maximize their benefits and minimize any possible harm to the patient. The application of digital health technologies considerably facilitates this task through the use of more available patient data, better medication review, and evidence-based treatment choice (Almontashiri et al., 2024).

Prescription and electronic health records allow for gaining full access to the patient’s medication history, laboratory test results, and other relevant clinical data. It makes it possible to optimize therapy by means of proper medication reconciliation, drug-related problem identification, and treatment regimen optimization. Moreover, digital health data facilitate cooperation between healthcare professionals and continuity of care in different medical facilities (Ajans Samuel et al., 2025).

Use of AI-driven tools makes it possible to identify complicated medication use patterns and predict possible drug-related problems. AI-assisted tools may help pharmacists identify high-risk patients, predict drug complications, and make evidence-based recommendations for treatment. AI-assisted medication management is especially useful in treating complicated patients with several conditions and taking many drugs (Adeyemi et al., 2024).

Remote monitoring technologies also help in the process of medication optimization because of the possibility of ongoing assessment of the effectiveness of treatment and adherence. The ability to monitor continuously makes it possible for the clinical pharmacist to make necessary changes to the treatment plan right away (Awala and Olutimehin, 2024).

### 4.2 Clinical decision support and medication safety

The modern clinical pharmacy practice involves the use of clinical decision support systems, which are tools that help clinicians make medication decisions on the basis of patient-specific clinical information. The use of CDSS systems makes it possible for clinical pharmacists to detect possible drug-drug interactions, dosing mistakes, duplicate therapy, contraindications, adverse effects, and other information to provide better medication safety and prescribing quality (Wu et al., 2025).

The introduction of CDSS systems into the workflow of the healthcare facility has led to substantial improvement in the practices of medication safety in both inpatient and outpatient settings (Insani et al., 2025). The use of AI in clinical decision support makes it possible to introduce more sophisticated risk stratification models into the clinical decision-making process and to identify cases of patients at a high risk of being harmed due to medication. Clinical pharmacists play a vital part in translating information provided by CDSS into action plans in the clinical setting. Despite all developments in technology, the knowledge of pharmacists is indispensable when it comes to interpretation and translation of recommendations (Moon et al., 2024; Peiffer-Smadja et al., 2024).

### 4.3 Pharmacovigilance and adverse drug reaction monitoring

Pharmacovigilance is one of the crucial aspects of the clinical pharmacist’s work that involves detecting, evaluating, analyzing, and preventing adverse drug reactions and medication-induced harm. Digital technologies are widely used to enhance the efficiency of pharmacovigilance by facilitating ADR detection, reporting, and analysis (Komi et al., 2025). Electronic health record systems and digital reporting tools allow faster and easier identification and documentation of suspected ADRs. These innovations allow better signal detection through the systematic accumulation and analysis of medication safety information among numerous patients. Digital reporting systems also contribute to more effective communication between healthcare specialists and the pharmacovigilance system, which results in faster ADR signal detection (Gomase, 2025).

Artificial intelligence also contributes to the field of pharmacovigilance through better and faster signal detection and analysis of huge datasets. Machine learning algorithms provide an opportunity to detect possible safety signals faster than using traditional methods in electronic health record systems, spontaneous reporting systems, and real-world clinical data (Ndagije et al., 2023). Clinical pharmacists play an important role in pharmacovigilance through the detection of suspected adverse drug reactions, evaluation of causality, reporting adverse events, and therapeutic changes to prevent patient harm (Konda, 2025).

### 4.4 Antimicrobial stewardship in digital healthcare

Antimicrobial stewardship is yet another important field in which digital health applications have made contributions toward expanding the role of clinical pharmacists. Antimicrobial stewardship is focused on optimizing antimicrobial therapy, improving clinical outcomes, reducing toxicity, and preventing antimicrobial resistance (Rawson et al., 2024).

Clinical pharmacists are vital participants in antimicrobial stewardship efforts through the process of antimicrobial selection, dosing, antimicrobial therapy monitoring, and de-escalation. Use of digital technology greatly facilitates the efforts of pharmacists in their stewardship work by providing better access to data regarding microbiology, prescription, and the particularities of patients’ antimicrobial therapy (Khan et al., 2025).

Clinical decision support systems may be used by pharmacists to assess the appropriateness of antimicrobial use, identify antimicrobial overprescribing, and guide evidence-based antimicrobial selection. The advanced AI systems can further help stewardship efforts by analyzing microbiology, clinical, and prescribing data to predict resistance patterns and optimize antimicrobial therapy (Lawal et al., 2025).

Digital health-assisted antimicrobial stewardship has great potential in enhancing infectious diseases management and decreasing the AMR burden in African healthcare systems. Nevertheless, it is necessary to enhance the existing digital infrastructure, improve access to diagnostic information, and develop the capacity of pharmacists for conducting antimicrobial stewardship (Rawson et al., 2024).

In summary, digital health and artificial intelligence are transforming clinical pharmacy practice significantly by providing opportunities to be more proactive, evidence-based, and patient-centered in managing medications. Technologies are further extending the scope of activities of clinical pharmacists in medication optimization, medication safety, pharmacovigilance, and antimicrobial stewardship to make them indispensable participants of digitally-enabled healthcare systems (Park et al., 2022; Silva et al., 2022).

## 5 Digital health and artificial intelligence in precision medicine in Africa

Precision medicine has become a game-changing development in modern healthcare since it involves changing the current paradigm of treatment from population-based towards a patient-centered one. The idea behind precision medicine is to combine genetic, molecular, clinical, environmental, and other patient-specific characteristics in order to prevent, diagnose, and treat diseases in a more accurate way. Contrary to conventional approaches, precision medicine involves tailoring healthcare interventions according to individual patients’ specific characteristics (Sarwar, 2023).

However, the quick growth of digital technologies and applications used in the healthcare sector, as well as in artificial intelligence, is considered one of the drivers that helps to introduce the advancements related to precision medicine at a fast pace (Allen, 2024). The use of digital technologies allows collecting and analyzing large-scale clinical and biological data, while AI contributes to the improvement of predictive analytics (Papachristou et al., 2024).

Moreover, in the case of Africa, the use of precision medicine can become the key opportunity to deal with various problems within its healthcare system and achieve successful results in providing effective treatment. African populations have the highest level of genetic diversity all around the world; thus, there appear to be many chances for the development of genomic medicine. However, there appear to be many challenges as well due to the differences in treatment responses of various groups of people (Sibomana, 2024).

### 5.1 Foundations of precision medicine in African healthcare

Precision medicine is based on the notion that there can be significant biological differences in patients with the same clinical diagnosis and, accordingly, they may respond differently to the same treatments. This may depend on various factors like genomics, nature of the disease, associated comorbidities, lifestyle, environment, etc (Edvardsson and Heenkenda, 2025).

The relevance of precision medicine in Africa is explained by the complexity of the disease burden and significant differences in the treatment response between individuals. Indeed, African countries have a double burden of both communicable and noncommunicable diseases like HIV/AIDS, tuberculosis, malaria, cancer, cardiovascular diseases, and diabetes. Thus, there is a need for the complex therapy of such conditions, which makes an individualized treatment approach critical (Ashinze et al., 2025; Liang et al., 2025; Sarwar, 2023).

Technologies related to digital health are crucial in the development of precision medicine as they help collect data, monitor patients, and integrate clinical information across healthcare systems. Technologies like electronic health records, remote monitoring solutions, and digital health platforms facilitate the availability of data and increase clinical insights, helping implement a precision medicine approach. Digital technologies become critically important in gathering longitudinal data on patients necessary for individualized care strategies (Edvardsson and Heenkenda, 2025).

Incorporating the concept of precision medicine into Africa’s healthcare sector will lead to improved diagnosis, better selection of treatment options, reduction of adverse reactions to medication, and increased efficiency of the healthcare system. Nevertheless, this success will largely depend on having access to reliable clinical and biological information (Allen, 2024).

However, the adoption of precision medicine in clinical practice in Africa cannot solely rely on technological advancements. It needs more work on building up genomic research capabilities, access to digital health technology platforms, and proper integration of clinical, laboratory, and genomic data. All this would be necessary for effective and equitable implementation of precision medicine (Ashinze et al., 2025).

### 5.2 Pharmacogenomics and genomic-informed care

Pharmacogenomics plays a key role in precision medicine as it deals with the way how genetic diversity affects drug metabolism, drug response, and susceptibility to adverse drug reactions. This approach offers a strong scientific base for personalized medicine, as it allows developing treatment methods based on individual patients’ genetic characteristics (Sarwar, 2023).

The significance of pharmacogenomics is especially high in the context of healthcare development in Africa because of the unique genetic diversity of the continent. Africans have numerous genomic variations affecting drug-metabolizing enzymes, transporters, and target molecules that can affect drug efficacy, toxicity, and even the choice of optimal dose in case of variants affecting CYP2D6, CYP2C19, CYP3A5, and VKORC1 genes (Edvardsson and Heenkenda, 2025; Sibomana, 2024).

Genomic-guided medical care is becoming increasingly important in the management of various health conditions such as oncology, cardiovascular disease, infectious diseases, and rare diseases. In the field of oncology, genomic-guided care may help to make the right choice of targeted treatment. Genomic approaches may be useful in choosing antiretroviral treatments with reduced toxic risk in case of infectious diseases (Thorat, 2025).

In spite of all the possibilities, there are still several problems with the use of pharmacogenomics in Africa. Namely, underrepresentation in genomic research, lack of access to genetic testing, and poor incorporation of the data into regular practices are the main ones. Genomic research continues to be conducted among European and North American samples, which makes the findings hard to apply to the situation in Africa. Therefore, the expansion of African genomics and increasing data accessibility are crucial for the implementation of precision medicine (Yalcouyé et al., 2026).

### 5.3 Artificial intelligence in precision medicine

AI technologies are one of the key tools used in precision medicine because of their ability to work with complex datasets of healthcare data and analyze them for the needs of the patients. The AI systems can work with extremely complex data that may include genomic data, clinical, imaging, biomarker data, and outcome data, which helps to characterize diseases better and choose the best treatment strategy (Nnaji, 2024).

The machine learning models have already proved to be quite efficient in finding biomarkers, classifying diseases, stratifying patients, and creating predictions. The mentioned capabilities are especially useful for precision oncology, genomic medicine, and chronic disease treatment (Akpara and Yinka-Banjo, 2024).

AI also facilitates the effective implementation of precision medicine in clinical practice through enhanced multimodal integration of data about patients from different aspects of their healthcare. The use of genomic, clinical, and real-world data by AI systems allows for more accurate analysis and provides more detailed information regarding the disease process and treatment response (Allen, 2024; Papachristou et al., 2024).

The application of AI-driven precision medicine in African healthcare environments presents significant opportunities for enhancing the provision of healthcare and its effectiveness. The ability of AI to enhance the analysis and interpretation of complex data and to overcome the problem of a lack of specialist personnel is of particular importance in this context (Ashinze et al., 2025).

Nonetheless, the successful implementation of AI-powered precision medicine in Africa is associated with the need for the availability of relevant datasets and the development of better infrastructure. Overcoming these barriers would allow for efficient deployment of precision medicine based on AI in Africa (Nnaji, 2024).

**FIGURE 2:**
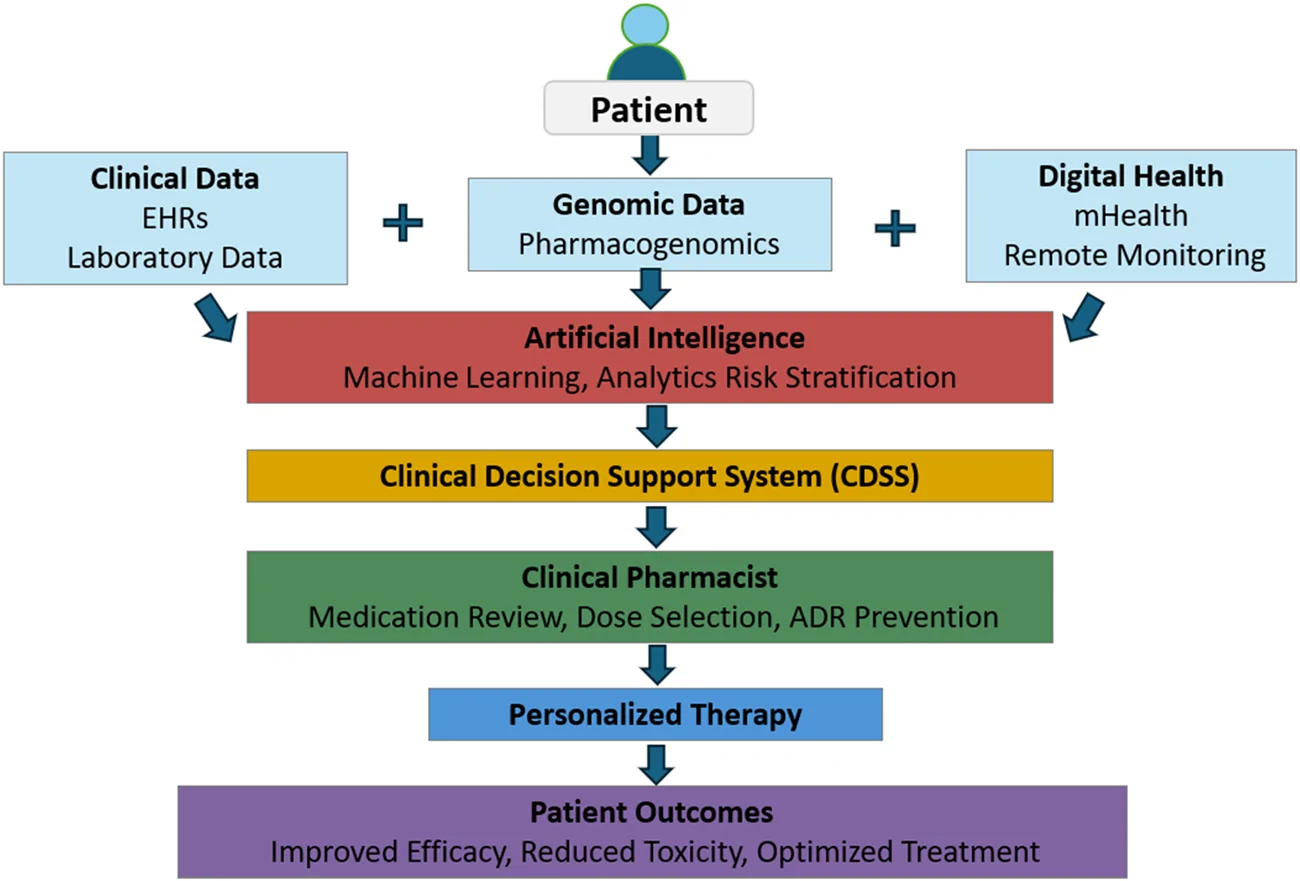
AI-enabled precision medication management workflow in African healthcare systems. Clinical, genomic, and digital health data are integrated using AI-based clinical decision support to enable personalized therapy and improve treatment outcomes.

Precision medicine in Africa would benefit significantly from the application of digital health and artificial intelligence due to the very high levels of genetic diversity on the continent. These tools facilitate the collection, integration, and analysis of big data that includes electronic health record data, mobile health data, wearable health data, and genomics data. The utilization of advanced machine learning algorithms for data processing allows for the improvement of patient risk stratification, disease prediction, treatment selection, and response to a particular medication (Roy et al., 2025). Additionally, the AI-enabled workflow for integrating clinical, genomic, and digital health information to support clinical decision-making and personalized therapy is illustrated by Figure 2.

As precision medicine becomes more popular in Africa, pharmacogenomic biomarkers would become a core component of the system of personalized healthcare. Table 3 summarizes major pharmacogenomic biomarkers and highlights their clinical relevance in African populations.

**TABLE 3:** Major pharmacogenomic biomarkers and their clinical relevance in African populations.

Gene/biomarker	Biological function	Drug examples	Clinical relevance	Implications in African populations	Major challenges	Key references
CYP2D6	Phase I drug metabolism enzyme	Codeine, Tramadol, Tamoxifen, Antidepressants	Determines metabolism of many commonly used drugs	High variability in allele distribution across African populations may result in poor or ultra-rapid metabolism, affecting efficacy and toxicity	Limited routine pharmacogenomic testing	Twesigomwe et al. (2023), Twesigomwe et al. (2025)
CYP2C19	Drug metabolism enzyme	Clopidogrel, PPIs, Antifungals	Influences drug activation and therapeutic response	Genetic variation may alter response to antiplatelet and gastrointestinal therapies	Underrepresentation in African pharmacogenomic studies	Booyse et al. (2023), Twesigomwe et al. (2025)
CYP3A5	Drug metabolism and clearance	Tacrolimus, Antihypertensives	Important in individualized dose adjustment	Common functional variants in African populations influence drug clearance and dosing requirements	Limited genotype-guided prescribing	Chamboko et al. (2023), Twesigomwe et al. (2025)
VKORC1	Vitamin K metabolism pathway	Warfarin	Determines anticoagulant sensitivity	Genetic variability contributes to major differences in warfarin dose requirements	Limited anticoagulation monitoring infrastructure	Twesigomwe et al. (2025)
SLCO1B1	Drug transport protein	Statins	Influences hepatic drug uptake	Variants may increase risk of statin-induced toxicity	Limited awareness in clinical practice	Haldar et al. (2025)
TPMT/NUDT15	Drug metabolism	Thiopurines	Predicts toxicity risk	Important for individualized dosing in oncology and autoimmune disorders	Limited implementation in Africa	Jarrar et al. (2025)
HLA Variants	Immune response regulation	Abacavir, Carbamazepine	Predict severe hypersensitivity reactions	Important in HIV and chronic disease management	Limited access to genomic screening	Banjoko et al. (2025), Misra et al. (2024)

## 6 Pharmacometrics and model-informed precision dosing in Africa

Pharmacometrics is a multi-disciplinary field that uses mathematical and statistical modeling to quantify, predict, and optimize the pharmacological effects and safety of drugs (Usman et al., 2023). Pharmacometrics combines information on pharmacokinetics (PK), pharmacodynamics (PD), physiology, and clinical data in order to support decision-making in drug development and application. Pharmacometric approaches help in the quantification of variability in drug behavior and response between patients, thus providing a basis for the optimization of treatment (Chotsiri et al., 2025).

Currently, pharmacometrics plays an important role in precision medicine in cases when there is a high degree of inter-patient heterogeneity. Such a situation is common for Africa due to its large genetic variability and the existence of differences in the distribution of diseases, nutritional status, environment, and availability of healthcare services. All this contributes to considerable variability in drug response, which makes conventional dosing regimens inefficient (Bandeira et al., 2023).

Thus, for African healthcare providers, pharmacometrics can be a valuable tool in achieving better dosing, reducing the risks of drug-related toxicity, and increasing the efficacy of treatment, especially in cases when there is a narrow therapeutic window or high pharmacokinetic variability of the medication (Adeagbo et al., 2026; Ndzamba et al., 2024).

### 6.1 Role of pharmacometrics in precision medicine

In personalized medicine, pharmacometrics serves as a tool for developing individual-based therapy optimization via modeling. As opposed to standard dosage regimens, the individual dose selection via pharmacometrics is based on specific factors affecting drug exposure and effect, such as age, body weight, organ system functions, disease severity, concomitant medications, and patient genotype (Marques et al., 2024).

One of the main fields in pharmacometrics is population pharmacokinetics (PopPK), where the interpatient variability in drug absorption, distribution, metabolism, and elimination is estimated. Moreover, in order to study the connection between concentration and effect of medication, the use of population pharmacodynamics (PopPD) models is implemented. The combination of PK and PD models allows quantification of the effect of treatment and provides a rationale for dose selection (Guidi et al., 2022).

The Model-Informed Precision Dosing (MIPD) is one of the most important clinical applications of pharmacometrics. This technique implies the creation of population models combined with information about specific patient characteristics (e.g., laboratory results, therapeutic drug monitoring, and clinical parameters). Bayesian forecasting allows even better personalization of the dose optimization process through continuous updates of dose predictions using patient-based data (Le et al., 2026).

In Africa, the use of pharmacometrics methods becomes especially relevant because the dosing regimen determined in other countries cannot always be suitable for an African patient. Genetic differences, characteristics of comorbidities, infectious diseases prevalence, nutrition, and even healthcare facilities can strongly influence the process of drug disposition and efficacy. Thus, the inclusion of African-specific information in PK/PD and MIPD models could help develop a more relevant dosing regimen (Ndzamba et al., 2024).

### 6.2 Clinical applications of pharmacometrics in African healthcare

The use of pharmacometrics has gained increased importance within healthcare systems in Africa, especially in diseases where there is a need for dose optimization and drug monitoring. The large variability of drug response in the African population makes pharmacometric modeling an appropriate technique that can be applied to achieve better treatment outcomes with low toxicity levels (Marques et al., 2024).

One of the key fields where pharmacometric modeling has found applications is in the management of infectious diseases, such as HIV, tuberculosis, and malaria, which continue to exert huge impacts on healthcare delivery in Africa (Abulfathi et al., 2022). Pharmacometric modeling has been extensively used in HIV management through the assessment of variability in drug exposure and the determination of specific patient doses. For instance, population pharmacokinetic modeling of antiretroviral drugs such as efavirenz and dolutegravir has indicated that there is large variability among patients in relation to body weight, age, medication, and genetic polymorphism (Liu et al., 2021).

Concerning tuberculosis, pharmacometrics is increasingly being used for dosing of anti-TB drugs. This includes first-line and second-line anti-TB drugs in cases of multidrug-resistant tuberculosis. In this case, the variability of drug exposure can affect the outcome of the treatment, emergence of resistance, and toxicity (Wilkins et al., 2022).

Malaria treatment also benefits from the application of pharmacometric techniques, especially in children and critical cases where traditional dosing does not always lead to adequate levels of the drug in the body. The development of population PK/PD models will assist in tailoring antimalarial treatments by taking into account physiological differences that are associated with age, disease severity, and local practices (Ncube et al., 2024; UMOH et al., 2024).

In addition to the treatment of infections, pharmacometrics also has its place in the field of oncology, as numerous anti-cancer drugs have a very narrow therapeutic index and toxicity concerns. Modeling-assisted dosing could assist in the formulation of personalized regimens that balance efficacy and toxicity (Darshit et al., 2026; Van der Heijden et al., 2023). Moreover, the issue of chronic diseases is also becoming increasingly important, and the pharmacometric guidance of dose optimization becomes an integral part of the treatment of hypertension, diabetes, cardiovascular disease, and antimicrobial stewardship.

The increasing problem of both communicable and non-communicable diseases in Africa creates the necessity to develop new, more personalized treatment approaches. A pharmacometric approach provides a way to achieve this goal (Liang et al., 2025; Sarwar, 2023). Pharmacometric applications in Africa are becoming more useful clinically within several treatment areas. The applications of pharmacometrics are most important in diseases that require personalized dose administration, therapy drug monitoring, and improved treatment effectiveness with reduced toxicity. The main pharmacometric applications and their clinical importance in Africa are shown in Table 4 below.

**TABLE 4:** Major clinical applications of pharmacometrics and model-informed precision dosing in African healthcare systems.

Clinical area	Key applications	Clinical benefits	AI/Digital integration	Examples in Africa	Major challenges	Key references
HIV management	Population PK modeling of antiretroviral therapy	Optimized dosing, reduced toxicity, improved viral suppression	EHR + AI-supported dose prediction	South Africa, Kenya, Uganda	Limited TDM and PK data	Liu et al. (2021)
Tuberculosis (TB)	PK/PD-guided dose optimization for anti-TB therapy	Improved efficacy, reduced resistance	Digital adherence monitoring	South Africa, Ethiopia	Limited monitoring infrastructure	Ndzamba et al. (2024)
Malaria	Antimalarial dose optimization	Reduced treatment failure	mHealth-based monitoring	Nigeria, Ghana, Tanzania	Pediatric variability	Ncube et al. (2024), UMOH et al. (2024)
Oncology	Precision dosing of chemotherapy	Reduced toxicity, improved efficacy	AI-assisted toxicity prediction	South Africa, Egypt	Limited oncology infrastructure	Darshit et al. (2026), Van der Heijden et al. (2023)
Antimicrobial stewardship	Model-informed antibiotic dosing	Better outcomes, reduced AMR	Clinical decision support systems	Emerging across Africa	Poor lab capacity	Khan et al. (2025), Rawson et al. (2024)
Chronic disease management	Dose optimization in cardiovascular and metabolic diseases	Improved disease control	Remote monitoring + wearables	Rwanda, Kenya	Fragmented long-term monitoring	Pillai et al. (2025), Usman et al. (2023)

The examples presented above illustrate the clinical usefulness of pharmacometric techniques. With improvement of digital health infrastructure and access to data, the use of pharmacometrics will keep expanding in clinical practice.

### 6.3 Integration of pharmacometrics with artificial intelligence and digital health

Pharmacometrics combined with AI and digital health has been reforming the future of precision medicine through dynamic, data-driven, and personalized therapy decision-making processes. In the conventional use of pharmacometric tools, the datasets used were mostly structured ones like clinical and pharmacokinetic data. But through the advancement of digital health technology, it is possible to incorporate patient-generated data in the form of wearable device output, electronic health records (EHRs), remote monitoring technology, and mobile health applications (Bandeira et al., 2023).

Through the use of AI in pharmacometrics, the efficiency of data processing, predictive analysis, and the performance of the model will be boosted. This can be done through machine learning algorithms that can discover non-linear relationships between the variables of the patient, treatment outcomes, and disease development, thus complementing the traditional pharmacometric methods (Ajans Samuel et al., 2025; Karalis, 2024).

For African healthcare systems, the combination of AI and digital health together with pharmacometric modeling opens new possibilities that allow overcoming resource constraints and enhancing therapeutic decision-making. The implementation of mobile health applications, telemedicine systems, and remote monitoring technologies would enable constant data collection, even in areas that lack appropriate geographic accessibility (Agbeyangi and Lukose, 2025). These technologies will help obtain real-world data for the creation of African pharmacometric models and personalized dosing approaches.

The use of AI-assisted CDSS might additionally increase the pace of adoption of such an approach, as the use of such systems will enable assistance to health professionals in real-time adjustments of dose and therapy optimization. For example, pharmacometric platforms equipped with AI technology could provide personalized antimicrobial dosing, chronic disease treatment and drug monitoring using the combination of patient profile information, laboratory results, pharmacogenomics data, and the course of treatment (Mbanugo, 2025; Tegegne et al., 2026).

Such an approach is especially relevant for the case of Africa, as the high inter-patient variability and challenges of the healthcare system require a new approach to medication management.

However, the successful integration of pharmacometrics, artificial intelligence, and digital health would need interoperable health IT infrastructure, good real-world data, and multi-disciplinary cooperation involving clinical pharmacists, physicians, pharmacometricians, and data scientists. If these needs are met, model-informed precision dosing in African healthcare systems would be more easily adopted.

### 6.4 Challenges of pharmacometrics implementation in Africa

In spite of the increasing opportunities for pharmacometric applications in precision medicine, there are several important barriers that limit large-scale implementation of such applications in the healthcare systems of Africa. One of the main barriers is associated with the lack of appropriate datasets collected on African patients. Currently, almost all pharmacometric models were created based on the data obtained from Europe, North America, and Asian countries, which cannot reflect the biological and clinical diversity of African patients (Janssen et al., 2022).

The infrastructure barrier plays an important role as well. Lack of sufficient laboratory facilities, TDM facilities, poor interoperability of digital health systems and electronic health record integrations become problems for many African healthcare institutions that do not have appropriate tools to provide the necessary real-time clinical data needed to apply pharmacometric analysis (Alimohamed et al., 2025).

Finally, the lack of experts in specific pharmacometric, clinical pharmacology, bioinformatics, and other areas also becomes an issue that complicates the process of implementation. The application of a pharmacometric approach in making clinical decisions requires interdisciplinary cooperation between clinicians, pharmacologists, pharmacometricians, data scientists, and other professionals. However, workforce capacity in these highly specialized areas is currently insufficient in many African countries. Financial and regulatory barriers further complicate implementation (Adeagbo et al., 2026; Pillai et al., 2025).

However, implementation of such pharmacometrics, artificial intelligence-based decision-support mechanisms, and digital infrastructure may require significant investments, which may not be possible in some regions. Furthermore, regulatory systems supporting the use of precision medicine, pharmacogenomics, and AI-based technologies in healthcare are insufficiently developed in the healthcare systems of many African nations (Ndzamba et al., 2024).

Solving such problems will require combined efforts to invest in infrastructure, develop professional skills, conduct biomedical research, and collaborate regionally. Conducting more African research in this field and including more Africans in clinical trials and pharmacometrics will be necessary to increase the reliability of pharmacometrics in the future.

## 7 Future perspectives

The future of the healthcare sector in Africa is now being determined by the integration of various innovative technologies in digital health, artificial intelligence, precision medicine, and pharmacometrics. The evolution of such rapidly developing spheres has opened up many possibilities for advancing healthcare accessibility, enhancing the quality of therapeutic decisions and medication management, and improving the overall outcome of patient care through different healthcare settings. The combination of these spheres can lead to revolutionary changes in the healthcare sector and significantly alter the provision of healthcare services (Alqahtani et al., 2025).

With the constant evolution of African healthcare systems, digital transformation becomes one of the most important factors of healthcare modernization. Together, digital health platforms, AI-based decision support systems, genomics, and quantitative pharmacometrics have all become very effective instruments that help advance the efficiency and effectiveness of healthcare. It is especially true in light of the numerous challenges that African healthcare systems are currently facing in terms of staff shortages, specialists’ unavailability, high disease burdens, and inadequate healthcare resources (Bandeira et al., 2023).

Despite the numerous barriers, the advancements in technology, computing power, biomedical research, and health innovations still open up new possibilities for achieving sustainable transformation.

### 7.1 Scaling AI-driven precision medicine in Africa

The application of artificial intelligence (AI) in precision medicine has great possibilities of changing the delivery of healthcare services in Africa through better diagnostics, risk prediction, patient stratification, and personalizing treatments. Additionally, AI is capable of processing and analyzing huge amounts of complex information using computational technologies, thereby facilitating clinical decision-making based on evidence from data analysis.

The major advantage of AI technology in the application of precision medicine is the capability of integrating multidimensional data sets in healthcare services, such as EHRs, laboratory test results, imaging findings, genomics, pharmacogenomics, and treatment outcomes. It allows the identification of clinically useful patterns in the collected information, which is helpful in biomarker discovery, disease classification, predictive modeling, and treatment planning. It is especially helpful in cases when there is high variability in treatment effects on patients, such as cancers, infectious diseases, heart-related illnesses, and chronic non-communicable diseases (Adeagbo et al., 2026; Ogunye et al., 2024). Precision medicine through the use of AI holds great promise in solving some of the existing healthcare problems in Africa, which include healthcare workforce shortage, lack of specialists, fragmented healthcare provision, late diagnosis, and high burden of diseases. Artificial intelligence clinical decision support systems will help improve the accuracy of diagnoses, optimize decision-making regarding treatments, and increase overall healthcare efficiency in general and specialized practices (Hussain et al., 2022).

The implementation of AI in the real-world setting has proved that AI innovations in healthcare provision in Africa are becoming increasingly feasible. AI-assisted diagnostics have shown promising capabilities in increasing the accuracy of disease diagnosis and speed of decision-making in resource-limited environments. AI-assisted tuberculosis screening, for example, through computer-aided chest radiography, is already being used in Ethiopia and South Africa to assist in earlier detection, increase screening, and enable treatment (Wilkins et al., 2022). Likewise, AI-enabled malaria diagnosis systems through smartphone-based microscopy and rapid diagnostic tests in Uganda and several West African countries have shown good potential through increased accuracy and reduced errors of human involvement (Ncube et al., 2024).

Continental approaches are also crucial in reinforcing Africa’s focus on digital transformation in healthcare. An example of such is the Africa CDC Digital Transformation Strategy (2023–2030), which forms a vital strategy for developing digital infrastructure, enhancing health information interoperability, improving disease surveillance systems, and fostering the adoption of AI in healthcare innovation among African Union member states. Such approaches will go a long way in scaling AI-enabled precision medicine as well as healthcare innovation in Africa (World Health Organization Regional Office for Africa, 2025).

While such an opportunity is evident on the continent, it must be understood that implementation of AI in precision medicine in Africa at scale will need significant investments in infrastructure, interoperable health information systems, computing resources, and data governance frameworks. Equally important will be the need for regulatory policies focusing on issues such as data privacy, cybersecurity, algorithm transparency, and ethical use of AI in healthcare (Mishra et al., 2026).

### 7.2 Development of genomic and pharmacometric models for Africa

The development of genomic and pharmacometric models for African populations is one of the most critical priorities for the advancement of precision medicine in Africa. Africa is a continent that boasts the highest genetic diversity among humans and presents great prospects for genomic research, biomarker discovery, and innovations in personalized therapy. At the same time, high genetic diversity makes for considerable inter-individual differences in disease susceptibility, metabolism, responsiveness to therapy, and the risk of toxicity (Abulfathi et al., 2022; Twesigomwe et al., 2025).

The development of specific models for pharmacogenomics and pharmacometrics among Africans is thus critical for improving individualized therapy and the equitable adoption of precision medicine. Population-specific models have been seen to improve dose customization, efficacy, and safety through considerations of different genetic backgrounds, disease loads, environmental exposures, and medical situations of different African populations (Bandeira et al., 2023).

**FIGURE 3:**
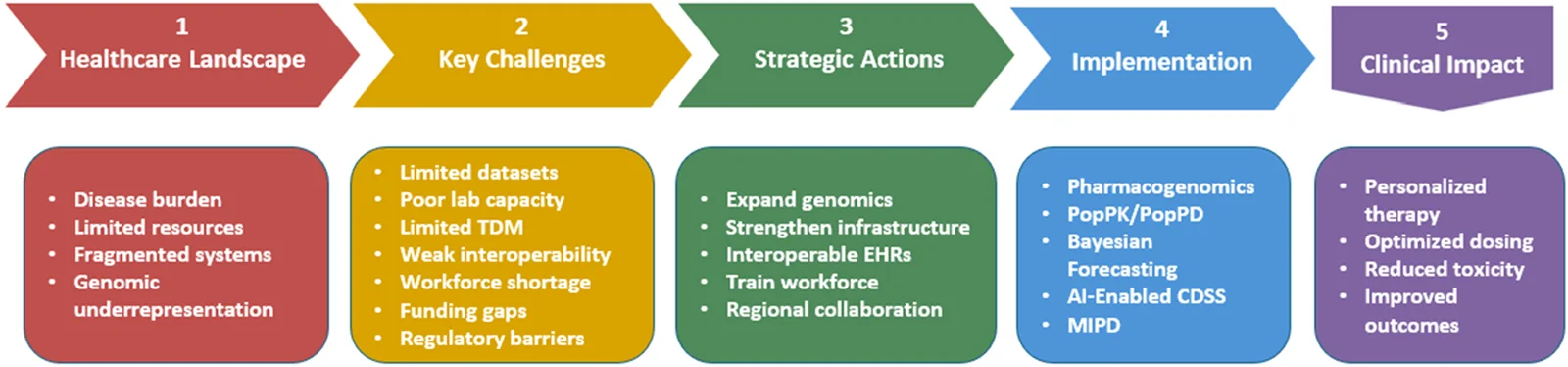
Roadmap for implementing pharmacometrics and model-informed precision dosing in African healthcare systems. TDM, therapeutic drug monitoring; EHRs, electronic health records; PopPK, population pharmacokinetics; PopPD, population pharmacodynamics; AI, artificial intelligence; CDSS, clinical decision support system; MIPD, model-informed precision dosing.

It will be vital to promote the establishment of genomic research initiatives, multicenter clinical trials, and pharmacometric research programs that can foster the creation of good locally relevant databases. Examples of such genomic research initiatives include the Human Heredity and Health in Africa (H3Africa) Consortium, which has been shown to make remarkable efforts in improving the research capacity and participation of Africans in genomics research. The overall strategic roadmap illustrating the transition from current barriers to successful implementation of pharmacometrics and precision dosing in African healthcare is presented in Figure 3.

## 8 Study strengths and limitations

This narrative review presents a comprehensive overview of the incorporation of digital health and artificial intelligence (AI) in African clinical pharmacy. Additionally, it focuses on challenges, opportunities, and implementation strategies in Africa, including recent evidence and current international and regional guidelines.

However, there are some limitations of this review. First, being a narrative review, it may be exposed to possible selection bias. Second, the existing literature from many African countries is still limited and heterogeneous due to the diversity of digital infrastructure and regulations in these countries. Third, the fast development of AI and digital health may limit the long-term applicability of some findings.

## 9 Conclusion

Digital health, artificial intelligence, precision medicine, and pharmacometrics-driven therapeutics are transforming the landscape of healthcare delivery and creating new possibilities for the future of healthcare in a more personalized and data-driven manner. Digital transformation in healthcare delivery in Africa has great potential to create new chances for improving the quality of healthcare, enhancing medication management, improving therapeutic efficacy, and reducing persistent disparities in healthcare delivery. Clinical pharmacists are supposed to have a significant role in this transformation. Their knowledge in such fields as pharmacovigilance, antimicrobial stewardship, therapeutic drug monitoring, and optimization of individual therapy makes them potential contributors to a digital revolution in precision healthcare delivery. With healthcare systems becoming more and more data-driven, the contribution of clinical pharmacists is anticipated to grow toward clinical decision-making and precision therapeutics.

However, there are several barriers, including a lack of infrastructure, staff, funding, data, and regulation. Nonetheless, there are also opportunities to speed up the process of implementation in healthcare delivery systems in Africa. In conclusion, there is great potential in the application of digital health, artificial intelligence, precision medicine, and pharmacometrics in clinical pharmacy practice that will help change healthcare delivery in Africa.
